# Supplementary material for: Mitochondrial-derived compartments buffer outer membrane protein load during acute mitochondrial adaptation
Source: bioRxiv. 2026 Jun 10:2026.06.09.731211. Preprint. [Version 1] doi: 10.64898/2026.06.09.731211 (PMC13278180; doi:10.64898/2026.06.09.731211)
Supplement: Supplementary file 1 [file NIHPP2026.06.09.731211v1-supplement-4.pdf]

bioRxiv preprint doi: https://doi.org/10.64898/2026.06.09.731211; this version posted June 10, 2026. The copyright holder for this preprint (which was not certified by peer review) is the author/funder, who has granted bioRxiv a license to display the preprint in perpetuity. It is made available under a CC-BY 4.0 International license.

Supplemental Figure Legends

Figure S1: Characterization of MDCs induced by mitochondrial biogenesis–activating conditions (related to Figure 1)

A. Immunoblot time course of mitochondrial protein levels in wild-type cells grown in glucose-replete conditions (YPAD) over 0-6 h. Por1, Om45, Aco1, and Cit1 are shown; Vph1 and Ponceau staining serve as loading controls.

B. Quantification of MDC diameter under different induction conditions. Wild-type diploid cells were treated with glucose restriction (YPA), rapamycin (RAP), or concanamycin A (ConcA).

C. Widefield images of MDC cargo colocalization following 2 h glucose restriction. Cells express Tom70–mCherry to label MDCs and the indicated GFP-tagged proteins (Tim50, Tcd2, Mcp1, Cox7, Oac1, or Dic1). White arrows indicate MDCs. Scale bar, 5 µm.

D. Quantification of MDC cargo colocalization, as shown in (C). Colocalization was scored when MDCs overlapped with the indicated GFP signal.

E. Immunoblot analysis of GFP processing for MDC cargo proteins following glucose restriction for the indicated times. Strains expressing Mir1-GFP, Tcd2-GFP, or Dic1-GFP were analyzed. Free GFP indicates proteolytic cleavage; GFP was detected by immunoblot, and Vph1 and Ponceau staining serve as loading controls.

F. Quantification of MDC formation following 2 h treatment with 2-deoxy-D-glucose (2-DG) at the indicated concentrations.

bioRxiv preprint doi: https://doi.org/10.64898/2026.06.09.731211; this version posted June 10, 2026. The copyright holder for this preprint (which was not certified by peer review) is the author/funder, who has granted bioRxiv a license to display the preprint in perpetuity. It is made available under a CC-BY 4.0 International license.

Quantification and statistics. For MDC frequency measurements, n = 3 independent experiments with 100 cells scored per replicate; for diameter measurements (B), n = 100 MDCs per condition. Error bars represent mean ± SEM.

Figure S2: Further characterization of the trace-glucose requirement and amino acid independence of MDC formation (related to Figure 2)

A. Quantification of MDC formation following 3 h carbon-source shifts in synthetic media. Cells were grown overnight in synthetic media with 2% glucose and shifted to media containing glucose (Glc), no added carbon source (-), glycerol (Glyc), galactose (Gal), sucrose (Suc), raffinose (Raf), or fructose (Fru).

B. Quantification of the percentage of cells exhibiting fragmented mitochondria (see 2B and 2D).

C. Quantification of MDC formation following 2 h cycloheximide (CHX) treatment under varying glucose and amino acid conditions. Cells were grown overnight in synthetic media and shifted to the indicated conditions.

D. Quantification of mitochondrial fragmentation in wild-type and *dnm1Δ* cells under different media conditions. Cells were grown overnight in YPAD and shifted for 2 h to YPAD, synthetic media with 0.02% glucose (S + 0.02% glucose), synthetic media without carbon (S), or synthetic media with glycerol (S + glycerol).

E. Quantification of MDC formation in wild-type and *dnm1Δ* cells under the conditions described in (D).

F. Analysis of whole-cell amino acid metabolites following 2 h treatments (YPAD, YPA, YPAG, S), shown as linear fold change relative to YPAD.

bioRxiv preprint doi: https://doi.org/10.64898/2026.06.09.731211; this version posted June 10, 2026. The copyright holder for this preprint (which was not certified by peer review) is the author/funder, who has granted bioRxiv a license to display the preprint in perpetuity. It is made available under a CC-BY 4.0 International license.

G. Quantification of MDC formation under trace-glucose conditions with or without amino acid supplementation. Prototrophic cells were grown overnight in YPAD, synthetic (SD), or minimal media and shifted for 2 h to the indicated conditions, including glucose restriction (0.02% glucose) and casamino acid supplementation.

H. Quantification of MDC formation under trace-glucose conditions in minimal or nitrogen-starvation media. Cells were grown overnight in minimal media with 2% glucose and shifted for 2 h to the indicated conditions.

Quantification and statistics. Microscopy-based assays (A–E, G–H) were performed with n = 3 independent experiments and 100 cells scored per replicate. Error bars represent mean ± SEM. Statistical analysis for (F) was performed using two-way ANOVA with Holm–Šídák multiple-comparisons testing; false discovery rate (FDR) correction (q = 0.05) was applied.

Figure S3: Snf1 is specifically and acutely required for MDC formation induced by mitochondrial biogenesis–activating conditions (related to Figure 3)

A. Quantification of MDC formation in PKA-pathway mutants following 2 h glucose restriction.

B. Quantification of MDC formation in Snf1-associated mutants following 2 h glucose restriction.

C. Immunoblot analysis of Snf1-AID-FLAG strains and controls following auxin treatment. Haploid parent strains, an untagged OsTIR1 control, and diploid Snf1-AID-FLAG strains were treated with DMSO or auxin for the indicated times. FLAG was detected by immunoblot; Ponceau staining serves as a loading control.

bioRxiv preprint doi: https://doi.org/10.64898/2026.06.09.731211; this version posted June 10, 2026. The copyright holder for this preprint (which was not certified by peer review) is the author/funder, who has granted bioRxiv a license to display the preprint in perpetuity. It is made available under a CC-BY 4.0 International license.

D. Quantification of MDC formation in Snf1-AID strains following auxin-induced degradation and 2 h carbon-source switching to galactose. Cells were pretreated with DMSO or auxin for 30 min before the media shift.

E. Quantification of MDC formation in Snf1-AID strains following auxin treatment and 2 h under trace-glucose conditions. Cells were pretreated with DMSO or auxin for 30 min before the media shift.

F. Quantification of MDC formation in Snf1-AID strains following auxin treatment and 2 h exposure to 2-deoxy-D-glucose (2-DG). Cells were pretreated with DMSO or auxin for 30 min before 2-DG treatment.

G. Quantification of MDC formation in Snf1-AID strains following auxin treatment and 2 h concanamycin A (ConcA) exposure. Cells were pretreated with DMSO or auxin for 30 min before ConcA treatment.

H. Quantification of MDC formation following auxin pretreatment and glucose restriction. Control (Snf1-AID-FLAG + EV) and test (Snf1-AID-FLAG + His3-OsTIR1 + *SCM4* OE) strains were grown overnight in YPAD, pretreated with DMSO or auxin for 30 min, and subjected to 2 h glucose restriction.

I. Quantification of MDC formation in EV control, SCM4 OE, *snf1Δ*, and *snf1Δ* + *SCM4* OE strains following 2 h glucose restriction.

Quantification and statistics. All MDC measurements were performed with n = 3 independent experiments and 100 cells scored per replicate. Error bars represent mean ± SEM. Statistical significance was assessed using Welch's two-tailed t-test.

bioRxiv preprint doi: https://doi.org/10.64898/2026.06.09.731211; this version posted June 10, 2026. The copyright holder for this preprint (which was not certified by peer review) is the author/funder, who has granted bioRxiv a license to display the preprint in perpetuity. It is made available under a CC-BY 4.0 International license.

Figure S4: Analysis of Snf1-dependent pathways involved in MDC induction (related to Figure 4)

A. Quantification of MDC formation in mutants affecting Snf1-regulated and mitochondrial remodeling pathways following 2 h glucose restriction.

B. Quantification of MDC formation following auxin pretreatment and glucose restriction. Control (Acc1-AID–FLAG) and test (Acc1-AID–FLAG + His3-OsTIR1) strains were grown overnight in YPAD, pretreated with DMSO or auxin for 30 min, and subjected to 2 h glucose restriction.

C. Quantification of MDC formation in Acc1-AID strains following auxin treatment and 2 h rapamycin exposure. Cells were pretreated with DMSO or auxin for 30 min before rapamycin treatment.

D. Quantification of MDC formation under combined treatments with DMSO, rapamycin (RAP), concanamycin A (ConcA), YPAD (control), or YPA (glucose restriction), with or without cerulenin pretreatment. Cells were pretreated with DMSO or cerulenin for 30 min followed by 2 h treatment.

E. Immunoblot analysis of AID strains used in Figure 4F and G and Figure S4B-C following auxin-induced degradation. FLAG was detected by immunoblot; Ponceau staining serves as a loading control. The asterisk indicates a nonspecific band.

F. Quantification of MDC formation in CRISPR-generated Mig1 phospho-null mutants following 2 h glucose restriction. Haploid mutants were mated with *mig1Δ* strains prior to analysis.

bioRxiv preprint doi: https://doi.org/10.64898/2026.06.09.731211; this version posted June 10, 2026. The copyright holder for this preprint (which was not certified by peer review) is the author/funder, who has granted bioRxiv a license to display the preprint in perpetuity. It is made available under a CC-BY 4.0 International license.

G. Immunoblot analysis of mitochondrial protein levels in wild-type cells following glucose restriction or NaCl stress in the presence of DMSO or thiolutin. Om45 and Por1 are shown; Vph1 and Ponceau staining serve as loading controls.

Quantification and statistics. All MDC measurements were performed with n = 3 independent experiments and 100 cells scored per replicate. Error bars represent mean ± SEM. Statistical significance was assessed using Welch's two-tailed t-test.

Figure S5: Mitochondrial protein expression and MDC regulation during distinct induction conditions (related to Figure 5)

A. Immunoblot analysis of mitochondrial protein levels in wild-type cells following 6 h treatment with rapamycin, ConcA, CHX, YPA, or S. Om45 and Por1 are shown; Vph1 and Ponceau staining serve as loading controls.

B. Immunoblot analysis of mitochondrial protein levels in Snf1-AID–FLAG strains following glucose restriction or rapamycin treatment with or without auxin-induced degradation. Om45 and Por1 are shown; Vph1 and Ponceau staining serve as loading controls.

C. Quantification of MDC formation in Hap4 expression strains following rapamycin treatment. Cells were grown overnight in SD-URA with or without β-estradiol to induce sustained Hap4-FLAG expression and then treated with rapamycin for 2 h.

Quantification and statistics. MDC measurements in (C) were performed with n = 3 independent experiments and 100 cells scored per replicate. Error bars represent mean ± SEM. Statistical significance was assessed using Welch's two-tailed t-test.

bioRxiv preprint doi: https://doi.org/10.64898/2026.06.09.731211; this version posted June 10, 2026. The copyright holder for this preprint (which was not certified by peer review) is the author/funder, who has granted bioRxiv a license to display the preprint in perpetuity. It is made available under a CC-BY 4.0 International license.

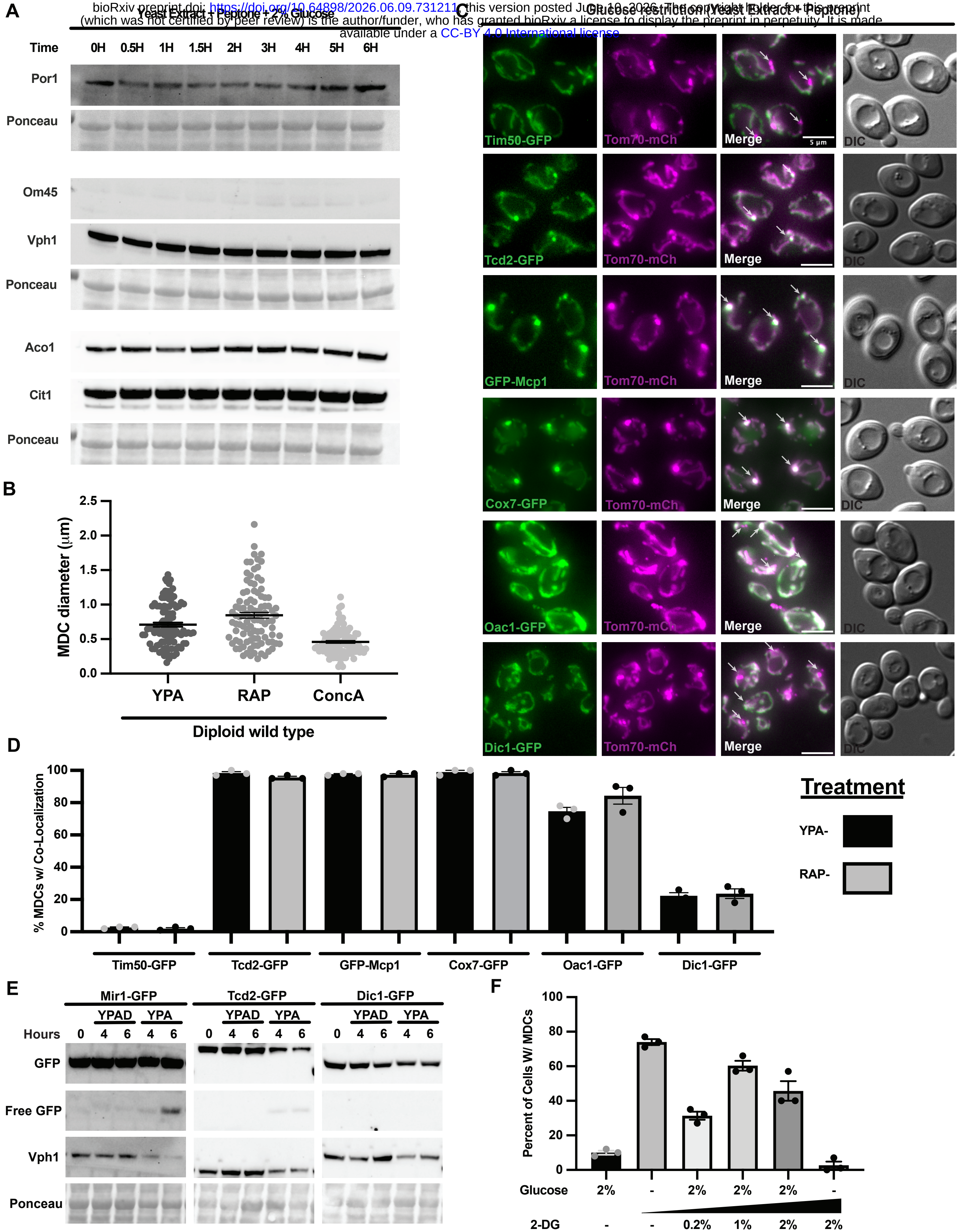

Figure S1

bioRxiv preprint doi: https://doi.org/10.64898/2026.06.09.731211; this version posted June 10, 2026. The copyright holder for this preprint (which was not certified by peer review) is the author/funder, who has granted bioRxiv a license to display the preprint in perpetuity. It is made available under a CC-BY 4.0 International license.

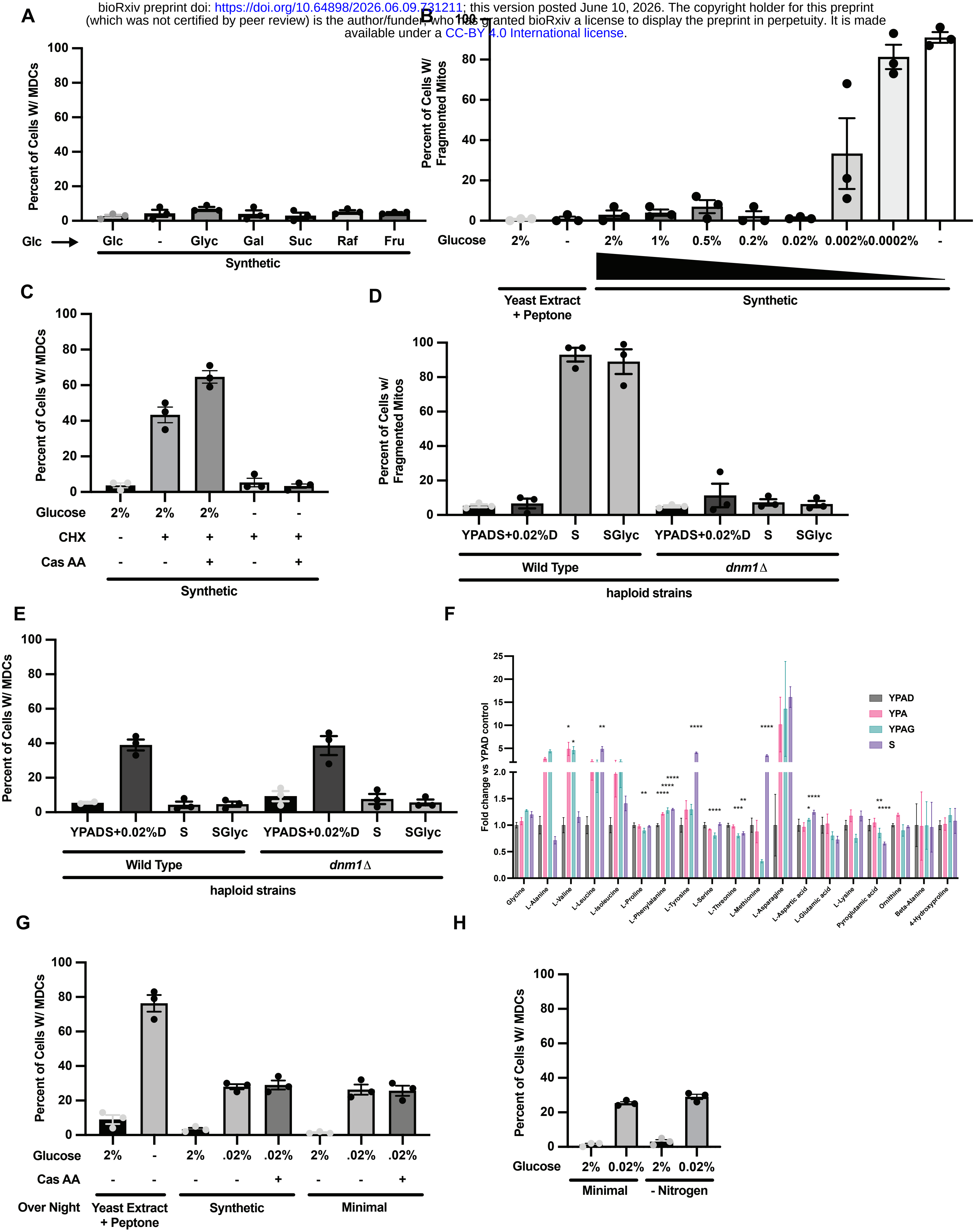

Figure S2

bioRxiv preprint doi: https://doi.org/10.64898/2026.06.09.731211; this version posted June 10, 2026. The copyright holder for this preprint (which was not certified by peer review) is the author/funder, who has granted bioRxiv a license to display the preprint in perpetuity. It is made available under a CC-BY 4.0 International license.

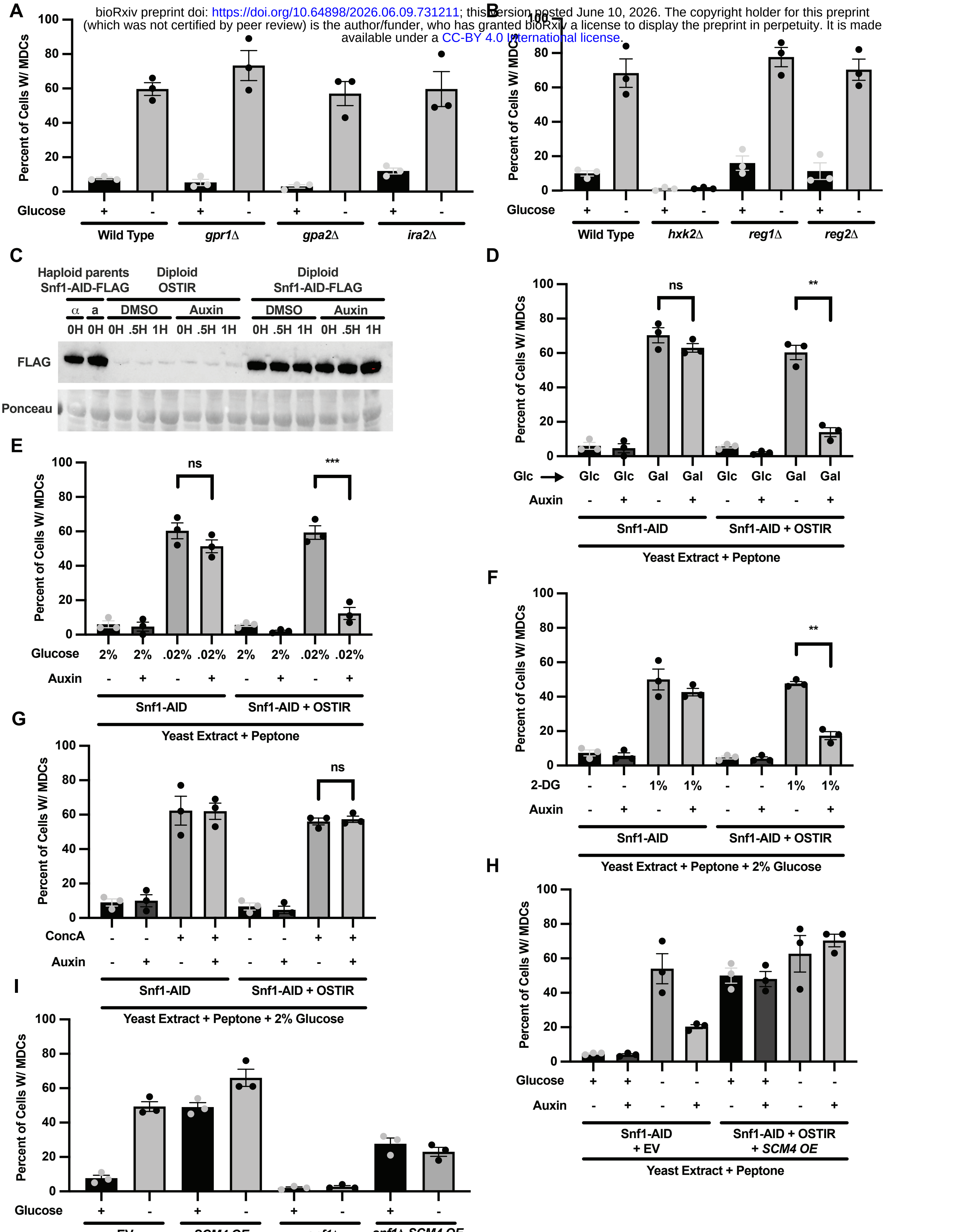

Figure S3

bioRxiv preprint doi: https://doi.org/10.64898/2026.06.09.731211; this version posted June 10, 2026. The copyright holder for this preprint (which was not certified by peer review) is the author/funder, who has granted bioRxiv a license to display the preprint in perpetuity. It is made available under a CC-BY 4.0 International license.

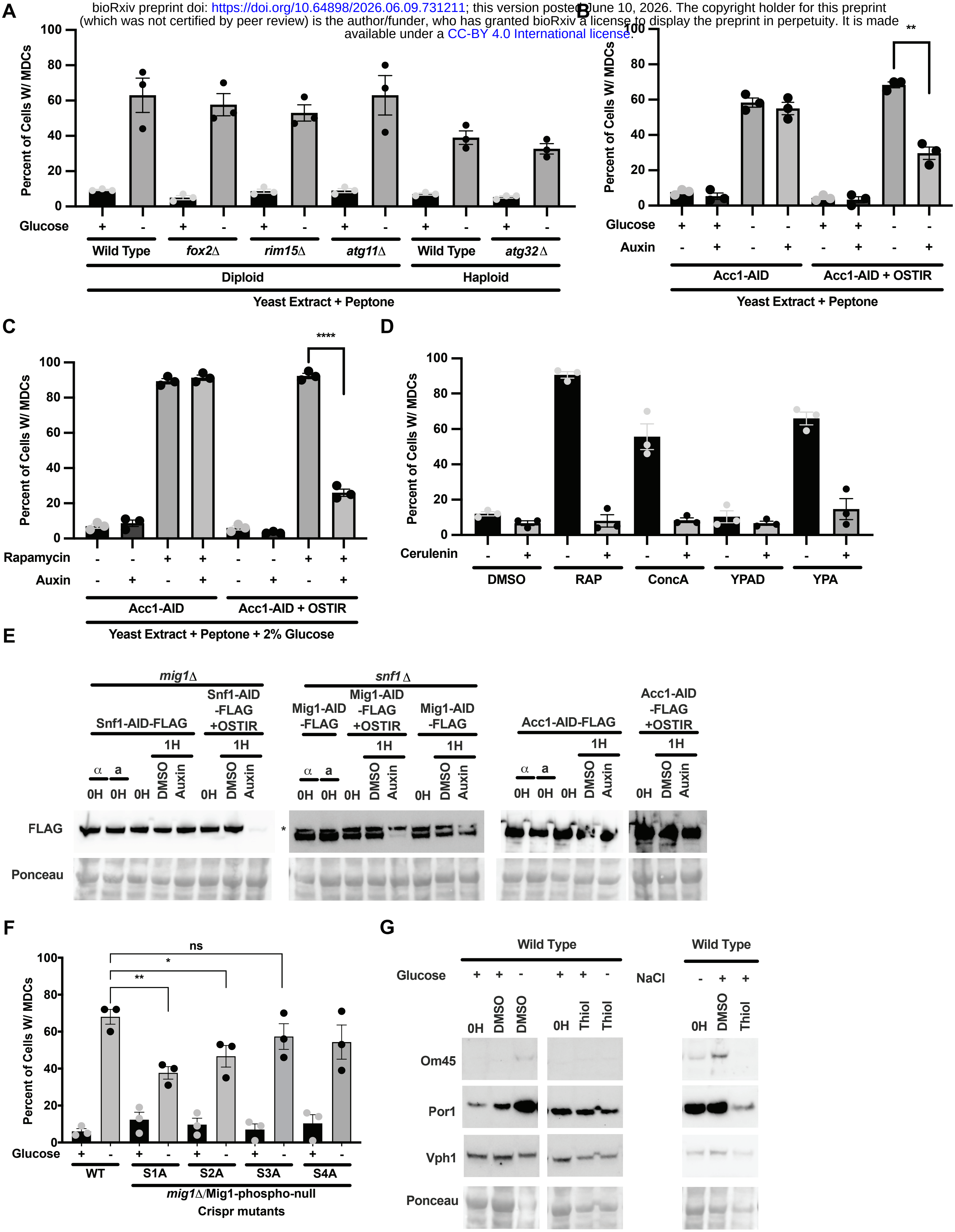

Figure S4

bioRxiv preprint doi: https://doi.org/10.64898/2026.06.09.731211; this version posted June 10, 2026. The copyright holder for this preprint (which was not certified by peer review) is the author/funder, who has granted bioRxiv a license to display the preprint in perpetuity. It is made available under a CC-BY 4.0 International license.

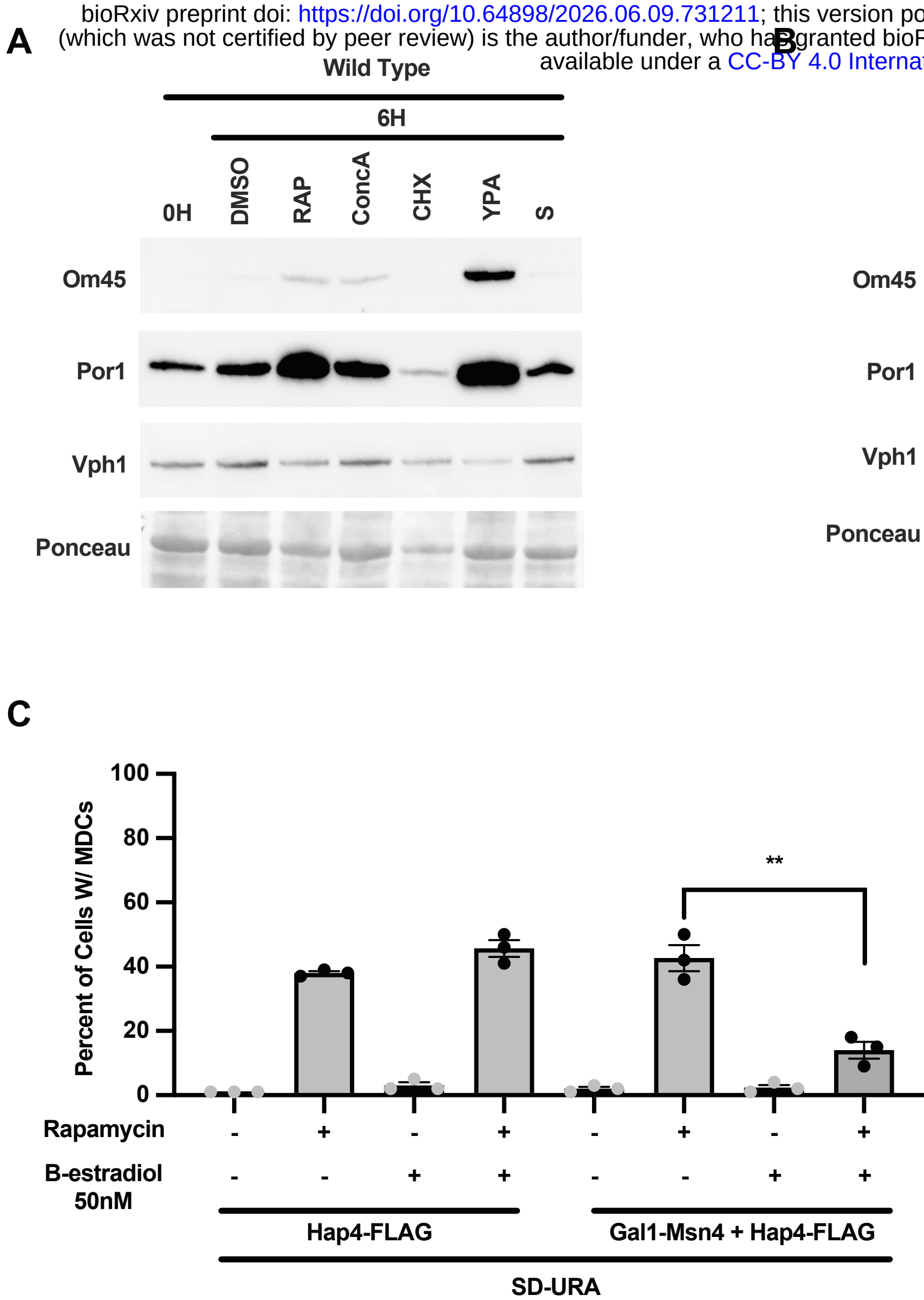

Figure S5
